# Role of platelet biomarkers in inflammatory response

**DOI:** 10.1186/s40364-020-00207-2

**Published:** 2020-08-02

**Authors:** Yufei Chen, Haoxuan Zhong, Yikai Zhao, Xinping Luo, Wen Gao

**Affiliations:** Department of Cardiology, Huashan Hospital, Fudan University, No.12 Middle Wulumuqi Road, Jing’an District, Shanghai, 200040 China

**Keywords:** Platelet, Inflammatory response, Immune regulation, Activation-related biomarkers

## Abstract

Beyond hemostasis, thrombosis and wound healing, it is becoming increasingly clear that platelets play an integral role in inflammatory response and immune regulation. Platelets recognize pathogenic microorganisms and secrete various immunoregulatory cytokines and chemokines, thus facilitating a variety of immune effects and regulatory functions. In this review, we discuss recent advances in signaling of platelet activation-related biomarkers in inflammatory settings and application prospects to apply for disease diagnosis and treatment.

## Introduction

Platelets were first reported to be involved in hemostasis, thrombosis and wound healing. However, there is accumulating evidence that platelets also have distinct roles in inflammatory response and immune regulation [1, 2]. This stems from several platelet characteristics, including the ability to bind infectious pathogens, secrete various immunoregulatory cytokines and chemokines, and express receptors for various immune effects and regulatory functions [3]. In addition, although platelets have no nucleus, using messenger RNA (mRNA) as templates, they can synthesize a limited number of proteins under different environmental pressures and transport inflammatory substances to inflammatory cells [4].

Platelets are discoid-shaped fragments generated from megakaryocytes in the bone marrow, which change to a compact sphere with dendritic extensions facilitating adhesion on activation. A steady supply is secured by a continuous production and clearance of 10^11^ platelets per day to maintain 150–400*10^9^/L of blood level [5]. Thus, numerous platelets may encounter pathogens, which are immediately bound following their intrusion to prevent pathogen dissemination and protect from infections. In fact, adverse clinical outcomes in septic patients are correlated with the decline of platelet count [6]. Observational studies also suggested that antiplatelet therapy was associated with a decrease in mortality from sepsis, without causing an excessive bleeding [7]. In line with this, platelet transfusions improved survival in mouse sepsis models [8, 9]. It is becoming increasingly clear that platelets are multifunctional and critical in many other physiological and pathological processes, such as inflammatory response and immune regulation [1, 10–14].

The association between platelets and inflammation has been evaluated for decades in the study of atherosclerosis [15]. Atherosclerosis is a chronic inflammatory process, and platelets interact with white blood cells and endothelial cells to promote inflammatory response in atherosclerosis. Platelet activation, adhesion to endothelial cells, and secretion of inflammatory molecules support the migration and adhesion of monocytes to the lesion and accelerate atherosclerosis progress. This review highlights these non-hemostatic aspects of platelets in inflammation and immunity, and their application prospect as candidates for targeted therapeutic approaches.

## Platelets and pathogens

Platelets can carry pathogens, including viruses, bacteria and parasites, on their plasma membrane and internally [11, 16–18]. Platelets are also known to be involved in acute and chronic liver disease associated with hepatitis B virus (HBV) by upregulating migration of virus-specific CD8+ T cells and non-specific inflammatory cells into the liver [19]. In contrast, platelets directly eliminate *Escherichia coli (E. coli)*, particularly when the bacteria are opsonized by IgG [20]. Two groups recently reported that platelets and megakaryocytes, precursor cells of platelets, engulf influenza virus and dengue virus, respectively [21, 22]. Generally, the antimicrobial activity in platelets is dependent on the IgG receptor FcγRIIA and actin rearrangement processes [20]. Furthermore, the activated platelets adhere onto *Staphylococcus aureus (S. aureus)* by interaction of P-selectin with P-selectin glycoprotein ligand-1 (PSGL-1) of neutrophils in neutrophil extracellular traps (NETs), thus they can inhibit bacterial growth by secreting the antibacterial peptide β-defensins or involving in the high mobility group box 1 protein (HMGB1) signaling pathway [23–26]. Besides contributing to the elimination of pathogens directly and indirectly, activated platelets can also kill infected red blood cells via platelet factor (PF) 4- and Duffy Ag-dependent manner, thereby controlling the infection of *plasmodium falciparum* [11]. However, the mechanism of how platelets kill endogenous parasites is unclear. Thus, patients with platelet disorders consistently are more susceptible to infection, which justifies detailed examination of the role of platelets in inflammation and immune responses.

Nevertheless, the combination of infectious agents with platelets may lead to the spread of infection. During sepsis, platelet activation promotes the development of disseminated intravascular coagulation (DIC), which blocks blood vessels instead and increases ischemia and multiple organ failure [27]. Additionally, septic-induced platelet activation upregulates both pro-inflammatory and anti-inflammatory cytokine networks [28]. Platelets bind to neutrophils and release NETs, which is beneficial in trapping bacteria. Yet it can be harmful to the host as a double-edged sword. For instance, when the neutrophils activated by LPS-bearing platelets, they are significantly activated and release NETs with reactive oxygen species, causing damage to the underlying endothelial cells [25]. In addition, neutrophils can scan activated platelets through the P-selectin ligand signaling pathway, leading to inflammation cascade [14]. At present, there is abundant evidence that platelets can act as pathogen sensors in peripheral circulation because they express several receptors that have no obvious effect on hemostasis [2].

## Platelet TLRs

Toll-like receptors (TLRs) are a major family of receptors that recognize pathogen-associated molecular patterns (PAMPs). Ligands of TLRs have been extensively studied, ranging from the secretory components of pathogens to nucleic acids. It has been demonstrated that platelets express TLRs 1–9, and some of them play a role as signal transducer, such as TLR4, which is a classical initiator of innate immune responses [29–31]. During sepsis, platelet TLR4 triggers the previously mentioned platelet-neutrophil interaction, which leads to NETs formation and subsequent bacterial capture [13]. It was indicated that platelets may be primarily responsible for reactivity to bacterial products and platelets may act as circulatory sentinels, binding to infectious agents and presenting them to neutrophils and/or cells of the reticuloendothelial system [32–34]. Numerous studies have also showed that activated TLRs and endotoxemia can increase the level of thrombopoietin (TPO) induced platelet-neutrophil aggregation in turn [35, 36]. Additionally, platelet TLR2 can recognize PAMPs in G+ bacteria, mycobacteria and fungi [37, 38]. Platelet TLR2 can promote the expression of P-selectin and GPIIb/IIIa integration, producing reactive oxygen species which may act directly on bacteria [39]. Additionally, stimulation of TLR7 leads to the expression of P-selectin and CD154, suggesting select α-granule release [40]. Other TLRs, like TLR9, also have functions in platelet activation and but their role in inflammation remains to be determined [41].

## Platelet CD40L (CD154)

Activated platelets also express CD40L (also known as CD154), a member of the tumor necrosis factor family [42]. Platelet CD40L can bind to the endothelial cell membrane and interact with CD40, triggering a variety of inflammatory reactions, leading to the local release of adhesion molecules, such as ICAM1, VCAM1, CCL2, etc. [42] Furthermore, platelets are known as the predominant source of soluble CD40L (sCD40L), which can induce vascular cells to express E-selectin and P-selectin and initiate the release of tissue factor and interleukin- (IL-) 6 [43]. CD40L^−/−^ mice have defect in thrombus formation, but infusion of recombinant sCD40L normalizes this deficiency, demonstrating the prothrombotic activity of sCD40L [42]. Therefore, platelet CD40L-CD40 axis may play a central link between thrombosis and inflammation.

Expression of CD40L on platelets has been shown to affect dendritic cells as well as B and T lymphocytes, suggesting that it provides a communicative link between innate and adaptive immunity [44–46]. For example, platelets inhibit dendritic cells differentiation in a CD40L-dependent manner and significantly suppress the proinflammatory cytokines production [47]. In addition, activated platelets enhance lymphocyte adhesion to endothelial cells and promote lymphocyte homing and migration to inflammatory areas [48, 49]. Besides, platelets expressed CD40L can promote B cell differentiation and Ab class switching [50]. Platelets can therefore modulate adaptive immune mechanisms through the CD40L-CD40 axis.

## Platelet MHC class I

Platelets always express major histocompatibility complex (MHC) class I, and MHC class I significantly increases during infection both on their plasma membrane and intracellularly [51, 52]. On the platelet plasma membrane, MHC class I appears unstable because molecules passively separated from platelets while stored in the blood bank, or eluted from the surface by chloroquine diphosphate or acid washing without affecting the integrity of platelet membranes [53]. At the allogeneic level, through transfusion, denatured platelet MHC class I interact erratically with CD8+ T cells (also known as CTLs) [54]. For example, the platelet MHC class I molecules themselves do not stimulate CTL-mediated cytotoxicity, but mediate “transfusion effect” known as the immunosuppressant response to the transfusion product [53]. Compared with non-transfused recipients, CBA mice transfused with allogeneic BALB/c platelets are easier to receive donor-specific skin grafts [55]. This observation supports the concept that allogeneic platelets may interfere with T cell-mediated cytotoxicity responses. Instead, recent study suggest that, platelet intracellular MHC class I molecules are connected with α granules and are mainly integral membrane proteins co-localized with β2-macroglobulin [56]. Now it seems that, activated platelets can express nascent MHC class I molecules, which have the ability to present antigens to CD8+ T cells. Thus, platelets can mediate T cell inhibition or activation, depending on the source of the MHC (on their plasma membrane or intracellularly).

## Platelet cytokines/chemokines

Platelets carry a number of chemokines and cytokines, which play substantial roles in various processes including hemostasis and wound repair, as well as pro-inflammatory and anti-inflammatory processes [57]. Platelets release various chemokines and cytokine upon activation such as CXCL1, PF4 (CXCL4), CXCL5, CXCL7, IL-8 (CXCL8), CXCL12, macrophage inflammatory protein- (MIP-) 1 α (CCL3), and RANTES (regulated on activation, normal T cell expressed and secreted, also called as CCL5) [58–60]. The major effect of these cytokines is to regulate inflammatory functions, like leukocyte migration, phagocytosis and reactive oxygen species (ROS) generation [61]. The most abundant chemokine is PF4, a positively charged protein that binds to glycosaminoglycans. PF4 not only has a role in haemostasis/thrombosis, but also is a chemotactic protein for monocytes and neutrophils, with immunoregulatory activity [62]. Interestingly, Guo et al. identify that PF4, a vital immunoregulatory chemokine, is essential for protecting mice against influenza A virus infection, especially as it affects the development of lung injury and neutrophil mobilization to the inflamed lung [63]. Moreover, PF4 prevents monocyte apoptosis, promotes monocyte differentiation into macrophages and induces phagocytosis and generation of ROS [64]. RANTES/CCL5 from platelets also play an important role in leukocyte recruitment, due to their potency to attract monocytes to inflamed endothelium [65], an effect that was found to be dependent on CCL5-receptor CCR1 [66]. Secreted RANTES form heterodimers to promote monocyte recruitment to the endothelium [67] by engaging its receptors CCR1 and CCR5, respectively [68–70].

In addition, platelets also synthesize and release IL-1β, IL-6, IL-8 and TNF-α in both micro vesicle and soluble form [71, 72]. When released upon platelet activation, IL-1β has been reported to mediate monocyte and leukocyte mobilization by binding to IL-1 receptors [73]. Furthermore, dengue virus infection could trigger IL-1β through the action of the nucleotide-binding domain, leucine-rich repeat containing protein 3 (NLRP3) inflammasomes in platelets [74]. Though without nucleus, platelets were recently reported to be competent for dengue virus replication, which may explain the thrombocytopenia associated with clinical dengue infection [75].

**Fig. 1:**
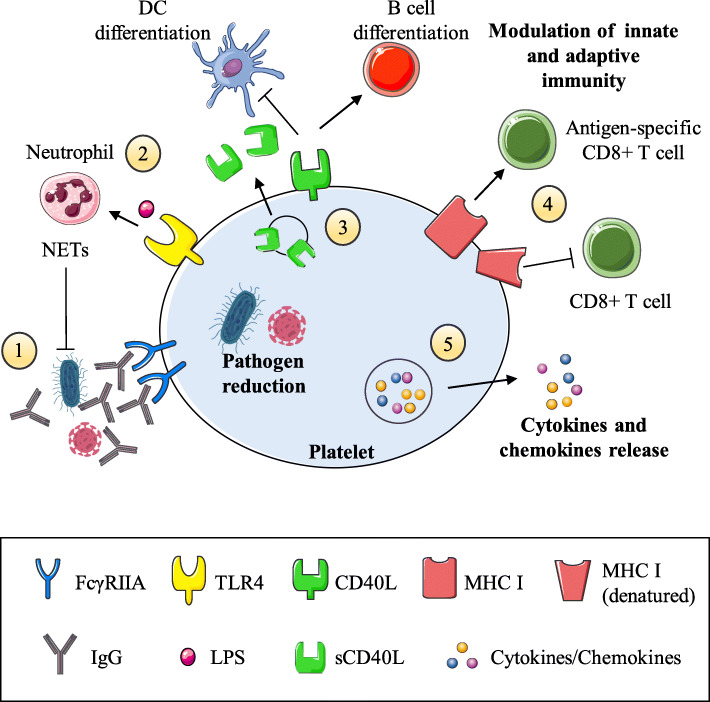
The key roles of platelets in modulating inflammatory processes. (1) Platelets are activated by invading pathogens (or their products) that have already been targeted by IgG receptor FcγRIIA (via IgG production). (2) Platelets can carry and eliminate pathogens, and via the expression of TLRs they can bind bacterial LPS and activate neutrophils, inducing NETs formation. (3) Platelet CD40L expression allows them to interact with different immune cells and either activate (arrow) and/or suppress (T bar) them. Furthermore, CD40L may be cleaved into a soluble form (sCD40L) that enhances platelet activation, aggregation, and platelet-leukocyte conjugation. (4) Intact platelet MHC class I molecules are located intracellularly but upon activation are expressed and can activate antigen-specific CD8+ T cells. In contrast, the MHC class I molecules on the surface of resting platelets are denatured and lead to CD8+ T cell inhibition. (5) Platelets contain many proinflammatory and anti-inflammatory cytokines and chemokines and, upon activation, can release them to the extracellular space. The culmination of these events makes platelets a formidable immunomodulatory host

A summary of all the above-mentioned characteristics is shown in Table 1 and illustrated in Fig. 1.

**Table 1:** Summary of major platelet mechanisms that modulate inflammation

**Pathogen Reduction**	
Carrying pathogens (viruses, bacteria and parasites) [11, 16–18]	
Elimination of viruses and bacteria [19–22]	
Inhibiting growth of *S. aureus* via β-defensins and NETs induction [23–26]	
Growth inhibition of plasmodia via PF 4- and Duffy Ag-dependent manner [11]	
**Platelet TLRs**	
Pathogen detection [1]	
TLR4: LPS-induced platelet-neutrophil aggregation [35, 36], bacterial trapping via NETs in sepsis [13], possible role in thrombopoiesis [29]	
TLR2: producing ROS which may act directly on bacteria [39]	
**Platelet CD40L (CD154)**	
Inflammatory reactions via interaction with CD40 of endothelial cells: release of adhesion molecules [42]	
Secreting soluble CD40L, and promoting thrombosis [42]	
Binding of DCs: inhibiting DC differentiation, suppressing the proinflammatory cytokines	
IL-12p70 and TNF-α, promoting IL-10 secretion [47]	
Triggering of T cell responses and migration to inflammatory areas [48, 49]	
Promoting B cell differentiation and Ab class switching [50]	
**Platelet MHC class I**	
Interference with T cell-mediated cytotoxicity responses [53–55]	
Intracellular MHC class I connection with α granules [56]	
**Platelet cytokines/chemokines**	
Carrying abundant chemokines and cytokines involved in pro/anti-inflammatory pathways [57]	
PF4: promoting monocytes and neutrophils migration [62], inducing leukocyte pro-inflammatory cytokine release, phagocytosis, chemotaxis, generation of ROS [64]	
RANTES (CCL5): promoting monocytes and macrophages chemotaxis and recruitment to the endothelium [65–70]	
IL-1β: central to pro-inflammatory cytokine cascade [73], possible role in dengue virus replication in platelets [74, 75]

## Clinical applications

In recent years, platelets have become important markers for various diseases. They are multifunctional blood particles, which may be candidates for innovative targeted therapeutic approaches. Besides playing a major role in physiological hemostasis, thrombosis and wound healing, platelets can also make great contributions to host inflammation and immune responses to infection and injury. Under uncontrolled pathological conditions, platelets play critical roles in acute coronary syndrome [76], central nervous system diseases [77], autoimmune diseases [78], and rheumatoid arthritis [79].

After activation, platelets secret numerous cytokines into peripheral circulation. Several of these cytokines, such as IL-1β, P-selectin, CD40L, PF4, and RANTES, are currently under consideration as molecular targets against inflammation and atherosclerosis [71, 80, 81]. Recently, the Canakinumab Anti-inflammatory Thrombosis Outcome Study (CANTOS) trial demonstrated a beneficial effect of the therapeutic monoclonal IL-1β antibody canakinumab on reducing the risk for recurrent cardiovascular events in high risk patients who have sustained a prior myocardial infarction [82]. The JAK/STAT pathway is recognized as one of the major mechanisms by which cytokine receptors transduce intracellular signals [83]. Numerous inflammatory immune-related diseases are driven by inflammatory mediators, which rely on JAK-STAT signaling. Therefore, inhibition of this pathway using JAK inhibitors might be a useful therapeutic strategy for these diseases. Tofacitinib, the first rheumatologic JAK inhibitor, is US Food and Drug Administration (FDA) approved for rheumatoid arthritis and is currently under investigation for other autoimmune diseases. TD-1473, a JAK inhibitor in phase II clinical trial of Crohn’s disease, exhibits inhibitory potencies in cellular assays similar to Tofacitinib [84]. Preclinical studies demonstrate that JAK inhibitor have potential suppression effects on platelet-derived growth factor in vascular smooth muscle [85] and antiplatelet effect [86], suggesting that JAK inhibition might be a viable strategy to treat multiple inflammatory response induced by platelets.

Moreover, due to short half-life of platelets, platelet-monocyte aggregates have emerged as new markers of platelet activation. Platelet-monocyte aggregates persist longer in peripheral blood and more sensitive in vivo than other platelet surface markers [80, 87].

Microparticles are extracellular vesicles produced by cytoplasmic vesiculation and division of cells ranging in diameter from 100 to 1000 nm. Platelets and megakaryocytes are the primary source of microparticles in circulation [88]. They can carry the nucleus and cytoplasmic components including proteins, lipids, and RNA from their precursor cells and transmit inflammatory signals to nearby or distant cells. Furthermore, given that they are induced in several inflammatory pathologies, such as atherosclerosis, stroke and autoimmune diseases, clinical applications of platelet-derived microparticles have been widely investigated [89–91]. Some medications, such as statins and aspirin, have been shown to reduce microparticles levels in patients and thus can be used to assess therapeutic efficacy [90, 92, 93]. Although important efforts in standardizing the preanalytical and analytical variables have been developed, standardization of microparticles detection and quantification methods is highly required to further confirm and generalize the results.

## Conclusion

Platelets are best known as primary mediators of hemostasis and thrombin generation; however, emerging evidence demonstrates that platelets are far more complex than previously regarded, equipped with elaborate intracellular machinery. Now recognized as key players in inflammation and immune responses, these anucleate cellular fragments express and secrete several pro- and anti-inflammatory molecules that serve to initiate and modulate immune functions. Understanding the signals of platelet biomarkers in inflammatory settings and how they modulate the immune system could expand new diagnostic and therapeutic methods to monitor and confront inflammatory immune-related diseases.

## Data Availability

Not applicable.

## Abbreviations

mRNA: Messenger RNA
HBV: Hepatitis B virus
*E. coli*: *Escherichia coli*
*S. aureus*: *Staphylococcus aureus*
PSGL-1: P-selectin glycoprotein ligand-1
NETs: Neutrophil extracellular traps
HMGB1: High mobility group box 1 protein
PF: Platelet factor
DIC: Disseminated intravascular coagulation
TLRs: Toll-like receptors
PAMPs: Pathogen-associated molecular patterns
TPO: Thrombopoietin
sCD40L: Soluble CD40L
MHC: Major histocompatibility complex
MIP: Macrophage inflammatory protein
RANTES: Regulated on activation, normal T cell expressed and secreted
ROS: Reactive oxygen species
NLRP3: Nucleotide-binding domain, leucine-rich repeat containing protein 3
